# MORA and EnsembleTFpredictor: An ensemble approach to reveal functional transcription factor regulatory networks

**DOI:** 10.1371/journal.pone.0294724

**Published:** 2023-11-30

**Authors:** Kevin Boyer, Louis Li, Tiandao Li, Bo Zhang, Guoyan Zhao

**Affiliations:** 1 Department of Neuroscience, Washington University School of Medicine, St. Louis, MO, United States of America; 2 Brown University, Providence, RI, United States of America; 3 Department of Developmental Biology, Washington University School of Medicine, St. Louis, MO, United States of America; 4 Department of Pathology and Immunology, Washington University School of Medicine, St. Louis, MO, United States of America; University of Illinois at Urbana-Champaign, UNITED STATES

## Abstract

**Motivation:**

Our study aimed to identify biologically relevant transcription factors (TFs) that control the expression of a set of co-expressed or co-regulated genes.

**Results:**

We developed a fully automated pipeline, Motif Over Representation Analysis (MORA), to detect enrichment of known TF binding motifs in any query sequences. MORA performed better than or comparable to five other TF-prediction tools as evaluated using hundreds of differentially expressed gene sets and ChIP-seq datasets derived from known TFs. Additionally, we developed EnsembleTFpredictor to harness the power of multiple TF-prediction tools to provide a list of functional TFs ranked by prediction confidence. When applied to the test datasets, EnsembleTFpredictor not only identified the target TF but also revealed many TFs known to cooperate with the target TF in the corresponding biological systems. MORA and EnsembleTFpredictor have been used in two publications, demonstrating their power in guiding experimental design and in revealing novel biological insights.

## 1 Introduction

Transcriptional regulation of gene expression is a fundamental biological process. It allows an organism to define cell identities during development, maintain normal tissue and cellular functions throughout its lifetime, and respond to various environmental stimuli. Transcription regulation is largely orchestrated by transcription factors (TFs) in collaboration with co-regulators and chromatin modifiers. TFs primarily regulate gene activity by binding to specific short DNA sequences, termed transcription factor binding sites (TFBSs), or motifs, located in the upstream, intron, or downstream regions of target genes. Mutations in TFs or TFBSs underlie many human diseases, such as cancer and neurological/neurodegenerative disorders [1, 2]. Understanding TF-mediated gene regulation can be instrumental in identifying the underlying disease mechanisms and potential targets for therapeutic development. We will use the term “transcription factor (TF)” specifically for proteins capable of binding DNA in a sequence-specific manner and regulating transcription. Proteins that are involved in transcription regulation but have no sequence binding specificity are referred to as “transcription regulators (TRs)”. TRs exert their regulatory function by interacting with TFs, modified histone protein tails, or methylated DNA sequences [3].

To understand how highly specific gene expression programs are orchestrated in complex organisms, we first need to characterize the TFs and their cognate TFBSs, which together determine the regulatory outputs of their target genes. The binding specificity of TFs is most commonly modeled using position weight matrices (PWMs) [4]. PWMs can be derived from aligning experimentally curated binding site sequences of a given TF, *in vitro* high-throughput SELEX (HT-SELEX) [5] and protein-binding microarray (PBM) [6] analysis, or *in vivo* technologies such as Chromatin Immuno-Precipitation sequencing (ChIP-seq) and CUT&RUN [7]. Typically, the identified DNA regions are long, and computational tools are required to determine the PWMs of targeted TFs and their actual binding sequences. Many databases have been created to provide the derived PWMs and their corresponding TFs, such as CIS-BP (catalog of inferred sequence binding preferences) [8], Factorbook [9], HOCOMOCO [10], JASPAR [11], HT-SELEX [12, 13], UniPROBE (the Universal PBM Resource for Oligonucleotide Binding Evaluation) [14], TRANSFAC [15], and HOMER [16]. CIS-BP is one of the most comprehensive publicly available motif databases which includes PWMs from most of the aforementioned databases [4]. PWMs can be used to scan any sequence to identify potential binding sites of the corresponding TFs, although this often leads to high false positive prediction rates due to the short (usually 6–20 bases) and degenerate nature of most TFBSs. However, the enrichment of TFBSs in a set of regulatory sequences has been proven useful in predicting biologically relevant TFs involved in regulating the expression of target genes [17].

Many computational tools have been developed to predict TFs and/or TRs that regulate a set of co-expressed or co-regulated genes. These tools can be classified largely into two categories based on the information they use to make predictions. The first category of tools performs motif enrichment analysis by comparing the distribution of TFBSs in a set of regulatory DNA regions to their distribution in a set of background sequences to determine the TFs with over-represented TFBSs. Some widely used tools in this category include HOMER [16], oPOSSUM-3 [18], Pscan [19], and Analysis of Motif Enrichment (AME) [20]. The second family of methods exploit experimentally determined protein-DNA binding profiles (such as histone marks and TF binding ChIP-seq data) and open chromatin information, herein referred to as (epi)genomic data, to identify candidate regulators. Example of tools in this category are the Binding Analysis for Regulation of Transcription (BART) [21] and Landscape In Silico deletion Analysis (Lisa) [22]. A common challenge faced by all these tools is how to prioritize candidate TFs for downstream biological mechanistic studies. Most tools predict hundreds of candidate TFs/TRs but lack a good indicator of biological relevance to prioritize these candidates since the ranking of candidates based on statistical significance does not necessarily reflect their biological relevance.

In this study, we developed two tools: MORA, a novel motif enrichment analysis tool, and EnsembleTFpredictor, an ensemble approach to provide a short-list of high-confidence candidate functional TFs by leveraging the predictive power of multiple TF-prediction tools. MORA utilizes CIS-BP, the most comprehensive motif PWM database currently available, and considers both motif density and motif abundance information to perform motif enrichment analysis. We used hundreds of datasets, including differentially expressed genes (DEGs) and ChIP-seq peaks regulated by known target TFs from human and mouse, to benchmark the performance of MORA and multiple state-of-the-art prediction tools. MORA outperformed all PWM-based tools (AME, HOMER, and Pscan) and was comparable to (epi)genomic-data-based tools (BART2 and Lisa2). EnsembleTFpredictor integrates the results from different TF enrichment tools to provide a list of candidate functional TFs ranked by prediction confidence. To our knowledge, this is the first tool performing this type of integration. Literature search and our experimental validation [23, 24] demonstrated that MORA and EnsembleTFpredictor are highly effective in identifying functional TFs responding to a perturbation and unraveling novel TFs that cooperate with each other to exert synchronized responses. Consequently, MORA and EnsembleTFpredictor serve as easy and cost-effective hypothesis-generating tools to broadly assess TF activity, yielding new biological insights. MORA is provided as a package composed of Perl and R scripts or a container image that can be run on a high-performance computing cluster or a single server independent of the computer operating system. EnsembleTFpredictor is provided as an R package and a web portal (https://github.com/GuoyanZhao-Lab/EnsembleTFpredictor).

## 2 Materials and methods

### 2.1 MORA implementation

**Fig 1:**
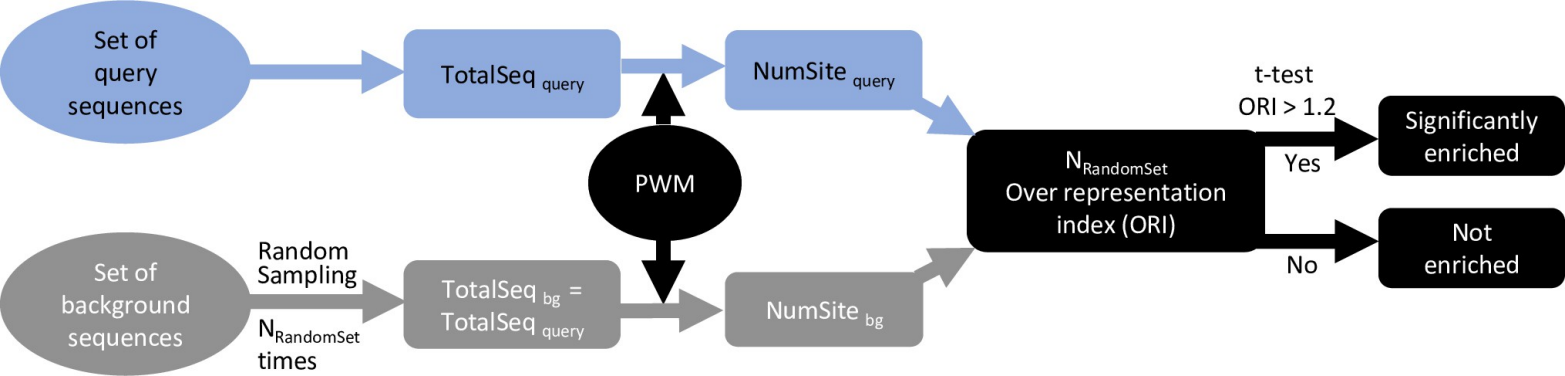
Schematic of MORA workflow.

MORA takes a set of query and background sequences in the FASTA format as inputs to perform motif enrichment analysis (**Fig 1**). The query sequences can be genomic regions upstream of user-defined gene sets (*e*.*g*., promoters) or putative DNA-regulatory regions derived from any genomic or epigenomic assays (*e*.*g*., peaks from ChIP-seq, CUT&RUN). In this context, we use the term “(epi)genomic data” to refer to both types of data. For gene-list analysis, genomic sequences up to 5-kb upstream of the ATG start codon of the query genes before reaching the next gene in the genome were retrieved and used as query sequences. The same genomic regions of genes that were not differentially expressed were used as the background sequences. For ChIP-seq peak analysis, genomic regions complementary to the query region were used as input to BEDTools [25] to generate random shuffled control sequence sets with the same length distribution as the query sequences. MORA initially randomly samples the background sequences to generate a set of sequences that have the same number and length distribution as the query sequences (herein referred to as “the control sequence set”). A user-specified number (N_RandomSet_) of control sequence sets is generated (100 by default). Given a PWM, the PATSER program [26, 27] is used to identify the putative binding sites in each sequence using the default cutoff score appropriate for the motif as determined by the probability of observing a subsequence of length L (L = length of the PWM) with a particular score or greater. With the information obtained, MORA calculates the over-representation index (ORI) using Formulas 1.1 and 1.2 [28, 29], comparing the query sequence set with each control sequence set for each PWM in the CIS-BP database. The ORI reflects the increased probability of finding a motif in the query sequence set compared to the control set, considering not only the number of motifs found in sequences but also the proportion of sequences in which the motif is found (Formula 1.1). A higher ORI indicates a greater enrichment of the motif in the query sequences compared to the control sequences, and an ORI higher than 1.2 was found to be effective in distinguishing enriched functional motifs [29]. A one-tailed Student’s t-test implemented in the *t*.*test* function of the R *stats* package was used to test the null hypothesis H0: μ > 1.2, where μ represents the mean of ORI. A PWM with a Bonferroni corrected p-value below 0.05 was considered statistically significant. Only PWMs present in at least 10% of query sequences were considered to represent biologically relevant TFBSs.


$$ORI=\frac{{Density}_{query}}{{Density}_{bg}}\times \frac{{Proportion}_{query}}{{Proporiton}_{bg}}$$ (1.1)

$$ORI=\frac{\frac{NumSite_{query}}{TotalLength_{query}}}{\frac{NumSite_{bg}}{TotalLength_{bg}}}\times \frac{\frac{N_{query}}{TotalSeq_{query}}}{\frac{N_{bg}}{TotalSeq_{bg}}}$$ (1.2)

NumSite _query_ is the number of sites of motif i found in query sequences;

NumSite _bg_ is the number of sites of motif i found in background sequences;

TotalLength _query_ is the total length of query sequences;

TotalLength _bg_ is the total length of background sequences;

N _query_ is the number of query sequences where motif i is found;

N _bg_ is the number of background sequences where motif i is found;

TotalSeq _query_ is the total number of query sequences;

TotalSeq _bg_ is the total number of background sequences.

MORA was implemented using the Perl and R programming languages. We provide MORA in three formats, each with an easy-to-use command-line interface: a Docker container image that is compatible with any computer operating system, a package designed for high-performance computing clusters using SLURM as a job management tool, and a package tailored for stand-alone Linux servers that supports multi-threading.

### 2.2 EnsembleTFpredictor implementation

**Fig 2:**
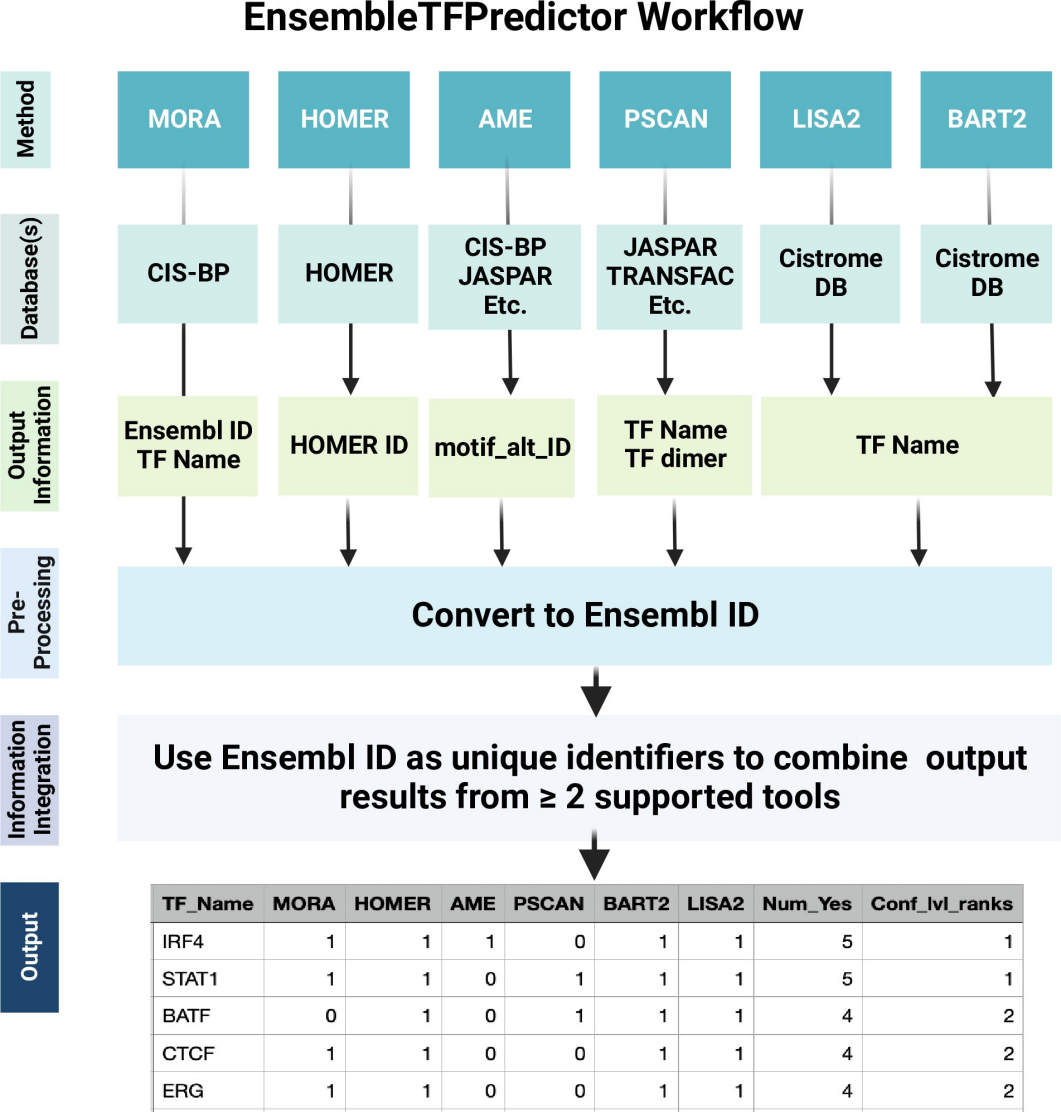
Schematic of EnsembleTFpredictor workflow. Created with BioRender.com.

Fig 2 depicts the workflow of EnsembleTFpredictor. The output files of MORA, BART2, and LISA2 include the names of predicted TFs, which can be directly used for downstream analysis. However, the output files of HOMER, AME, and Pscan have their own unique output formats without a shared identifier among the other tools. In some cases, TF names were embedded in database-specific identifiers or represented as dimers, which need to be pre-processed to extract the names of the predicted TFs. TF names were converted to Ensembl gene identifiers, which were used as standard names to ensure consistency across all tools. EnsembleTFpredictor integrates prediction results from all tools and ranks TFs based on the total number of tools predicting significant enrichment (**Fig 2**). EnsembleTFpredictor is provided as an R package and an R Shiny web-based interface to allow users without coding skills to perform the analysis.

### 2.3 CIS-BP database consolidation

CIS-BP 2.00 was downloaded from http://cisbp.ccbr.utoronto.ca. The database included 7704 PWMs for 1200 human TFs, 1158 PWMs for 938 mouse TFs, and 8 PWMs for 898 rat TFs. Only directly determined PWMs were included in the analysis. PWMs satisfying the statement "The maximum calculated p-value is less than the cutoff" as determined by PATSER were filtered out because of the lack of discriminative power. PWMs were consolidated because there is a many-to-many relationship between PWMs and TFs. For TFs with more than 3 PWMs, the top 3 PWMs with the highest information content per nucleotide were selected for downstream analyses. If the top PWM has an information content per nucleotide below 0.5, only the first PWM was selected for downstream analyses. The final consolidated high-quality CIS-BP database included a total of 3116 PWMs representing 1165 unique TFs (human, mouse, and rat genes with the same name were counted as one unique TF).

### 2.4 Benchmark datasets

To evaluate the performance of TF-prediction tools, we used two types of data where the identities of the functional TFs are known. The first type of data are published DEGs obtained from experiments in which the expression of specific TFs was perturbed using knockdown, knockout, overexpression, transgenic, or mutagenesis methods [22, 30]. This setting allows us to assess whether the target TF can be correctly identified. A total of 60 sets of DEGs for human (21 datasets) and mouse (39 dataset) were retrieved. First, gene names and gene expression fold changes for statistically significant DEGs reported in the publications were retrieved. Pseudogenes were removed from the gene lists. Next, the DEGs were divided into upregulated and downregulated gene sets. Genomic sequences up to 5,000 bp upstream of the ATG start codon or up to the next gene were retrieved in FASTA file format. The second type of data are TF ChIP-seq peaks downloaded from the ENCODE portal [31] (https://www.encodeproject.org/). A total of 512 ChIP-seq peak datasets for 337 unique TFs generated from the K562 human myeloid leukemic cell line were downloaded. BEDTools [25] was used to retrieve genomic sequences and generate random shuffled control sequence sets. Metadata for each dataset were downloaded and used to obtain target TF information.

### 2.5 AME, HOMER, Pscan, BART2, and Lisa2 analyses

We used the web version of AME for the analysis, employing the same FASTA sequence files used in the MORA analysis and the CIS-BP 2.00 database as the inputs. The “shuffled input sequences” option was selected for the control sequence. The corresponding species was selected, and the default settings were used for all other parameters. The reported adjusted p-value (below 0.05) was used to determine statistically significant PWMs. For the HOMER analysis of a list of genes, the same FASTA sequence files used in the MORA analysis were used as the query input. The upstream genomic sequences of all genes in the genome excluding the query genes were used as the background sequence. For ChIP-seq data analysis, background sequences were automatically selected by HOMER from the genome, matching for GC% content. A p-value of 0.1 or below was used as the statistical significance cutoff. The TF names were extracted from the unique HOMER PWM identifiers and used to obtain ENSEMBLE identifiers to link them to the TFs and TF family information in the CIS-BP database. The RefSeq identifiers of the query genes were used as the input for the Pscan analysis. The default -450 to +50 region in reference to the transcription start site of each gene was selected for all analyses. Pscan offered the JASPAR and the public release of TRANSFAC database, and the default Jaspar 2020_NR PWM database was selected for all analyses. The names of the 746 TFs from the Pscan output file were used as identifiers to be linked to the TFs and TF family information in the CIS-BP database. Dimers were manually separated to ensure the correct matching of TF names and the PWMs. A p-value threshold of 0.05 was used for statistical significance. A standalone local installation version of BART2 was used for all analyses. The “geneset” option was used for lists of genes as the input, and the “region” option was used for ChIP-seq peak input files. The appropriate species (human or mouse) was selected. The 7^th^ column of the ChIP-seq file was used as the score by BART2 to rank the peaks. A standalone local installation version of Lisa2 was used for the gene list analysis. The “FromGenes” interface of Lisa2 only requires a list of genes as input, with a minimum number of 15 genes. It is recommended to use 50 to 500 genes as input since using a small number of genes will lower the predictive power of chromatin profile modelling. The appropriate species (human or mouse) was selected.

## 3 Results

### 3.1 MORA implementation and performance evaluation

MORA compares the distribution of TFBSs predicted in a set of query sequences to the distribution in a set of background sequences to determine the TFBSs that are over-represented in the query sequences (**Fig 1**). The over-representation index (ORI) for each PWM in the consolidated CIS-BP database was calculated, taking into account both the number of motifs found in the sequences (motif density) and the proportion of sequences in which the motif is found (Formulas 1.1, 1.2). This approach is distinct from most motif enrichment methods, which assume either exactly one occurrence per sequence of the motif in the dataset (OOPS model) or zero or one motif occurrences per sequence (ZOOPS model) [17, 32]. Neither of these models considers the presence of clusters of the same motif in one sequence [17]. Homotypic clusters of TF binding sites (many adjacent TF binding sites for the same TF species) are prevalent in eukaryotic genomes and play an important role in gene regulation in human and other vertebrate genomes [33, 34]. A PWM with an ORI significantly higher than 1.2 is considered to be statistically enriched [29], and the corresponding TF represents the candidate regulator of the query genes.

**Fig 3:**
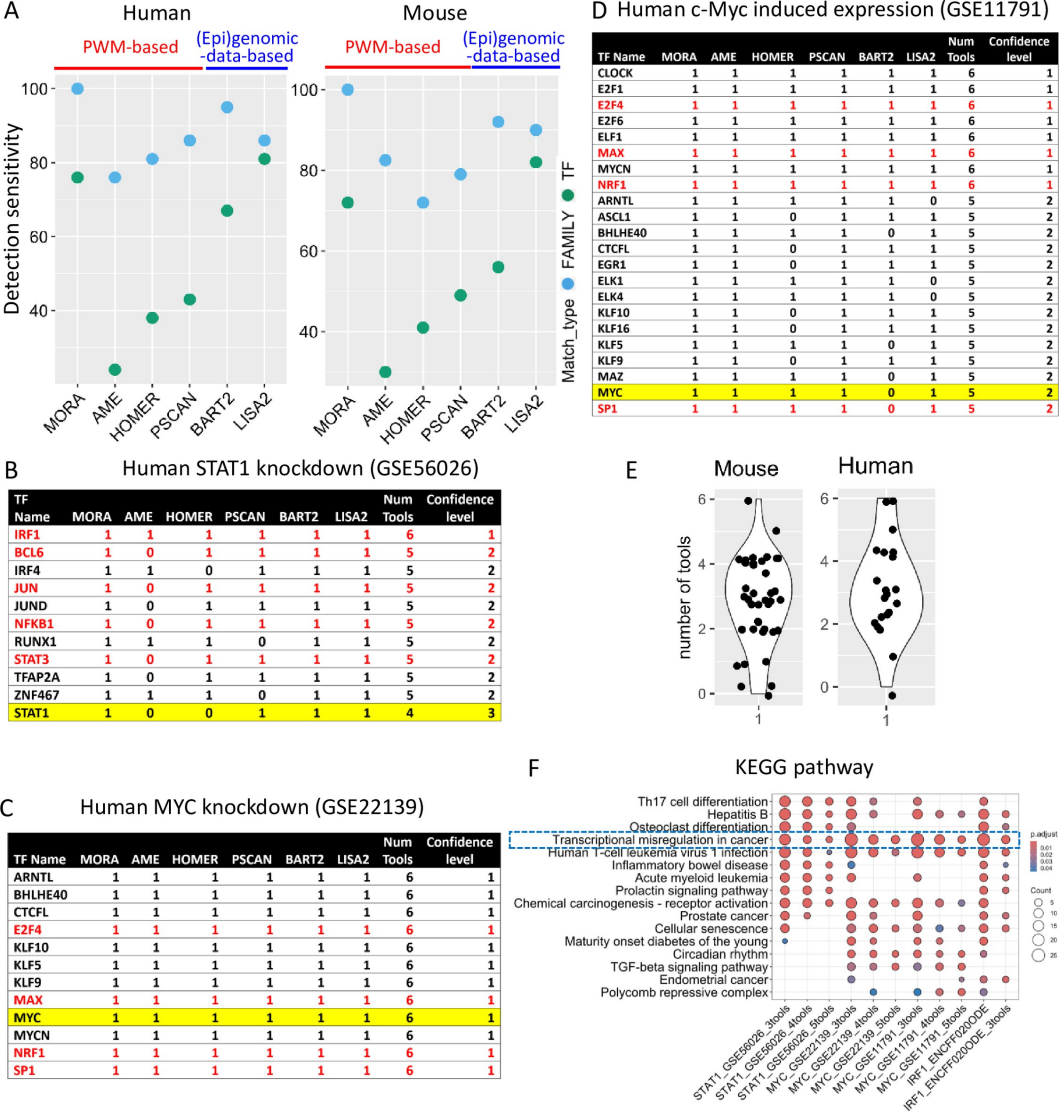
Performance evaluation of MORA and other published computational tools on gene lists. (A) Sensitivity plot of target TFs for human and mouse gene list data showing detection sensitivity of each method at both the TF and family level. (B-D) Ranked list of identified transcription regulators (TRs)/transcription factors (TFs) by prediction confidence level using EnsembleTFPredictor. The TFs highlighted yellow are the target TFs. Red font indicates TFs that are known to interact with the target TFs. (E) Violin plot showing the number of tools which correctly predicted the target TF from the gene list analyses for both human and mouse data. (F) TFs predicted by at least 3, 4, or 5 tools were enriched for KEGG pathways.

To systematically evaluate MORA, we compiled a benchmark panel of 60 DEG sets from 60 studies involving the knockdown, knockout, or overexpression of 15 unique target TFs in humans and 35 unique target TFs in mice (S1 and S2 Tables in S1 Data). MORA was separately applied to the upregulated and downregulated gene sets in each experiment because it has been reported that most PWMs of TFs are significantly over-represented in either upregulated or downregulated genes, but not in both [22, 30], which was also observed in the current study (S3 and S4 Tables in S1 Data). Therefore, we considered a TF to be correctly predicted if the PWM of the target TF was significantly enriched in at least one of the associated DEG sets. Prediction sensitivities were measured at both the TF level (exact match of the target TF) and the family level (if a TF within the same family as the target TF was predicted). In the human data, 16 out of 21 (76%) TFs were correctly predicted at the TF level, and all 21 (100%) TFs were correctly predicted at the family level (**Fig 3A**). In the mouse data, 72% (28/39) and 100% (39/39) of the TFs were correctly predicted at the TF and family levels, respectively. The 100% sensitivity at the family level suggested that MORA was able to identify TFs within the same family as the target TF, which tend to have similar binding specificities. Unfortunately, we were not able to evaluate prediction specificity due to a lack of knowledge regarding TFs that do not regulate the query genes.

### 3.2 Systematic performance comparison with previously published methods

Next, we compared MORA with multiple state-of-the-art TF-prediction tools (S5 Table in S1 Data), including both PWM-based tools (AME, HOMER, and Pscan) and (epi)genomic-data-based prediction tools (BART2 and Lisa2), when using a set of genes as the input. MORA is distinct from the other PWM-based tools in terms of motif enrichment measurements, TF binding motif site representation and searching algorithms, background sequences used for comparison, statistics tests, and PWM database (S5 Table in S1 Data).

For PWM-based tools, MORA outperformed AME, HOMER, and Pscan in terms of detection sensitivity. When using the same input sequence files and the same PWM database as in the MORA analysis, AME showed a sensitivity of 24% (5/21) at the TF level and 62% (16/21) at the family level for the human data (**Fig 3A and S6 and S7 Tables in S1 Data**). In the case of the mouse data, 12/39 (30%) and 33/39 (82.5%) were correctly predicted at the TF and family levels, respectively. HOMER uses its own PWM database, which includes 436 PWMs. HOMER reports the p-values as 1e0, 1e-1, 1e-2, etc. and recommends the use of a very strict p-value cutoff (*e*.*g*., 1e-10) to determine statistical significance. However, some target TFs were correctly predicted at a less stringent p-value cutoff of 1e-1. Because the most commonly used cutoff of p-value ≤ 0.05 was not reported, we used a p-value ≤ 0.1 as the statistical significance cutoff in order to recover more correctly predicted TFs. With this revised criteria, 8/21 (38%) and 17/21 (81%) were correctly predicted at the TF and family level respectively for the human data (**Fig 3A and S8 and S9 Tables in S1 Data**). A sensitivity of 41% (16/39) and 72% (28/39) were achieved at the TF and family levels, respectively, for the mouse dataset. Pscan differs from other tools in that it requires a text file with RefSeq IDs as input, and a region determined relative to the transcription start site (–450 +50 by default) of each gene is used in the analysis. Because the CIS-BP 2.00 database was not available as an option and the “User Defined” PWM database option was limited to a small number of custom PWMs, the default Jaspar 2020_NR PWM database was selected for all the analyses. A p-value < 0.05 was used as the cutoff for statistical significance. For the human data, 9/21 (43%) were correctly predicted at the TF level and 18/21 (86%) were correctly predicted at the family level (**Fig 3A and S10 and S11 Tables in S1 Data**). For the mouse data, 19/39 (49%) were correctly predicted at the TF level and 31/39 (79%) were correctly predicted at the family level.

MORA exhibits a sensitivity comparable to (epi)genomic-data-based tools. In terms of human data, BART2 showed a sensitivity of 67% (14/21) and 95% (20/21) at the TF and family levels, respectively (**Fig 3A and S12 and S13 Tables in S1 Data**), with one dataset failing to complete the analysis. Regarding mouse data, BART2 had a sensitivity of 59% (23/39) and 90% (35/39) at the TF and family levels, respectively. Lisa2 displayed a sensitivity of 81% (17/21) at the TF level and 85.7% (18/21) at the family level for the human dataset (**Fig 3A and S14 and S15 Tables in S1 Data**), with three query datasets failing the analysis. Concerning the mouse dataset, Lisa had a sensitivity of 82% (32/39) at the TF level and 89.7% (35/39) at the family level, with four query datasets producing no results for either upregulated or downregulated DEGs.

In summary, in terms of detection sensitivity, MORA outperformed all PWM-based tools both at the TF and family levels. BART2 and LISA2 belong to the category of tools that infer transcriptional regulators by integrating public chromatin accessibility and ChIP-seq data. Both tools outperformed AME, HOMER, and Pscan (**Fig 3A**) as reported [35]. MORA showed comparable performance to LISA2 and BART2, and it was the only tool with a sensitivity of 100% at the family level for both human and mouse datasets.

### 3.3 Ensemble approach to define high-confidence functional TFs and interactive regulatory networks

Experimental testing of candidate TFs for the regulation of a set of target genes can be labor-intensive, time consuming, and expensive. In most cases, each computational tool predicts hundreds of putative regulatory factors, and the statistical significance does not necessarily correlate with biological relevance. This makes it challenging to prioritize candidate TFs for downstream validation and mechanistic studies. On the other hand, metazoan TFs must, in general, work together with other TFs to achieve the required specificity in DNA binding and gene transcription control [3, 36, 37]. It has been shown that 10–15 TF binding sites (TFBS) are required to achieve the ~30 bits of information needed to target a specific gene in multicellular eukaryotes [36]. Therefore, predicted TFs, other than the target TF, likely include functional TFs that interact with the target TF in regulating the set of target genes. Because predictions tools differ in various aspects, including the fundamental mechanism of TF prediction (PWM enrichment vs. [epi]genomic-data modeling), the tested genomic regions, as well as the PWM databases and the statistical tests used (**S5 Table in S1 Data**), candidate TFs predicted by multiple tools are likely to represent biologically relevant regulatory TFs. Based on this rationale, we developed EnsembleTFpredictor to integrate the results of multiple prediction tools and provide a shortlist of high-confidence candidate functional TFs for downstream validation. Due to the use of different PWM or (epi)genomic databases by different tools, the identifiers of predicted TFs are not consistent across tools. We retrieved the unique identifiers of the predicted PWMs and converted the names of associated TFs to Ensembl IDs, which served as standard names for comparison across all tools (**Fig 2**). Because the complete set of TFs that regulate any given set of target sequences was not available to evaluate the prediction specificity, and the number of predicted TFs for each set of target sequences varied dramatically across tools, ranging from 0 to hundreds, we decided to assign equal weight to each tool. For each target gene dataset, EnsembleTFpredictor ranked the predicted TFs based on the number of tools that predicted the TF to be statistically significant and used this number as a measure of prediction confidence for each TF.

The performance evaluation of EnsembleTFpredictor on the set of DEGs demonstrates its efficacy in identifying not only the target TFs but also the regulatory TFs that cooperate with the target TFs in regulating gene expression. For example, a study (accession number, GSE56026) used microarrays to identify the changes in gene expression in a serous papillary endometrial cancer cell line treated with STAT1-siRNA [38]. STAT1 ranked third in terms of confidence level and was correctly predicted by 4 out of 6 tools (**Fig 3B and S16 Table in S1 Data**). Similarly, in a MYC knockdown experiment in medulloblastoma (GSE22139) [39] and in an induced Myc expression experiment in MCF-7 breast cancer cells (GSE11791) [40], MYC ranked first and second in the confidence levels and was correctly predicted by 6 or 5 out of 6 tools respectively (**Fig 3C and 3D and S19 and S20 Tables in S1 Data**). We summarized the information for all human and mouse target TFs and found that most TFs were correctly predicted by 2–4 tools (ranking 2–4, **Fig 3E and S17 and S18 Tables in S1 Data**) with only three target TF being correctly predicted by all the tools. One human TF and three mouse TFs were not predicted by any of the tools tested. Most target TFs (human 90.5%, mouse 84.6%) were correctly predicted by at least two methods. We therefore used the criterion of being predicted by at least two methods as the cutoff to determine whether a TF is a biologically relevant functional TF.

**Fig 4:**
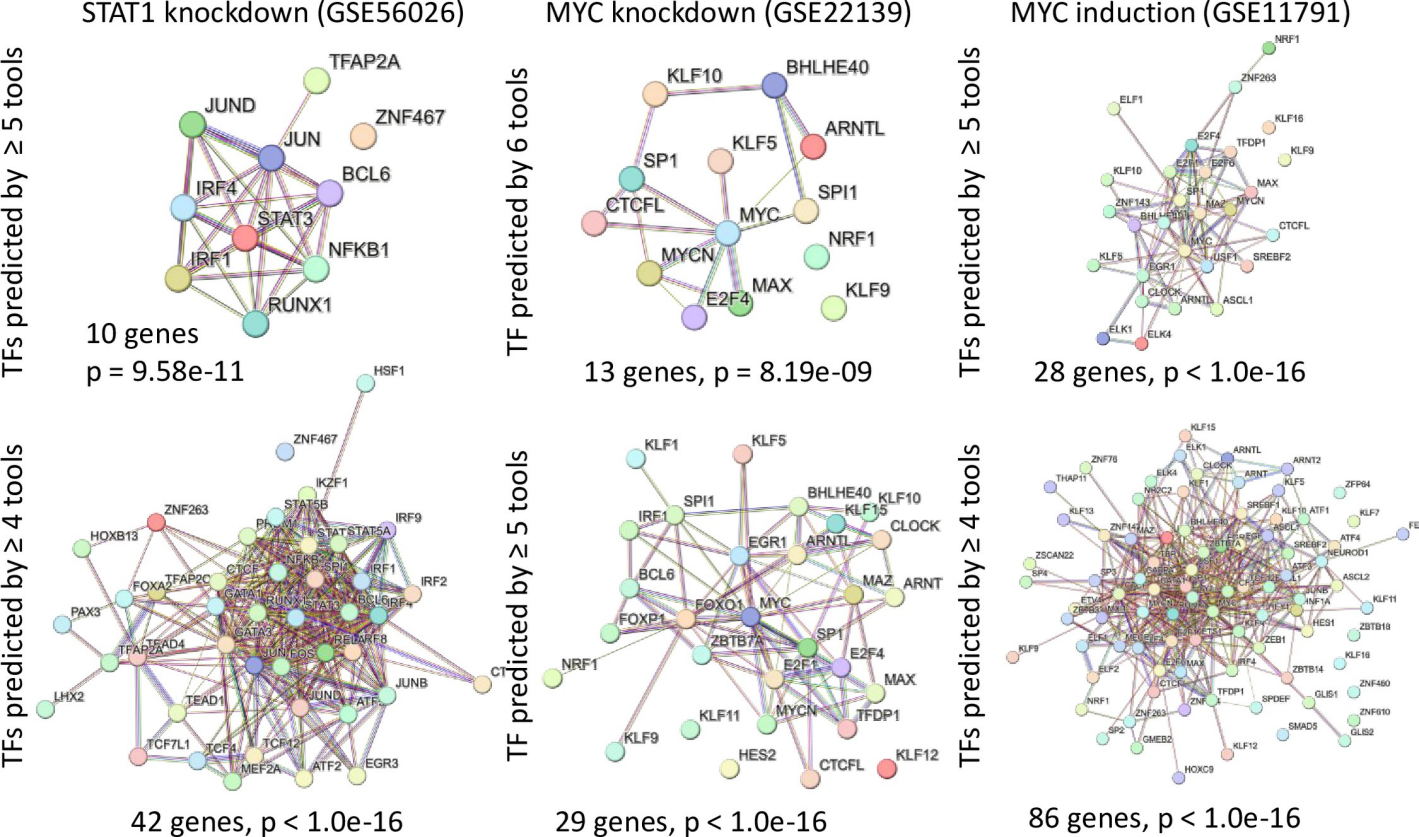
Protein-protein-interaction network analysis of TFs predicted by at least 4, 5, or 6 tools using STRING Database for gene list analysis.

Next, we examined the analysis results of query gene sets mentioned above to investigate the ability of EnsembleTFpredictor to identify TFs that interact with the target TFs. In the STAT1 knockdown data (GSE56026, **Fig 3B**), ten TFs, including BCL6, IRF1, IRF4, JUN, JUND, NFKB1, RUNX1, STAT3, TFAP2A, and ZNF467, ranked number 1 or 2 in confidence levels and were predicted to be statistically significant by ≥ 5 out of 6 tools. It is well established that STAT1, IRF1, and NFκB form regulatory networks by regulating each other’s synthesis or activation, or by converging at target promoters to cooperate or antagonize each other in the regulation of a common set of genes [41–43]. STAT3 and JUN cooperatively regulate transcriptional activation [44, 45], whereas STAT1 and BCL6 directly regulate each other’s expression in multiple biological contexts [46–48]. Therefore, most of the top-ranking TFs are biologically relevant TFs that cooperate with each other and the target TF to regulate gene expression. In the Myc datasets (GSE22139, GSE11791), multiple top-ranked TFs, such as MAX, SP1, NRF1, and E2F4, are known to interact with MYC in regulating the expression of common target genes in cancer cells [49–51] (**Fig 3C and 3D**). Next, we performed Kyoto Encyclopedia of Genes and Genomes (KEGG) pathway enrichment analyses using TFs predicted by at least 3, 4, or 5 tools as the query gene sets. “Transcriptional misregulation in cancer” was one of the top 2 enriched pathways for all datasets (**Fig 3F**) regardless of the cutoff used to determine the correctly predicted TFs. This is consistent with the fact that all three datasets were derived from cancer cell lines. We further evaluated protein-protein interactions among predicted TFs using the STRING Database [52] (https://string-db.org/). When using TFs predicted by at least 4 (42–86 genes), 5 (10–28 genes), or 6 (13 genes) tools as the query gene sets for the STRING Database search, we observed highly significant protein-protein interaction enrichment in all cases (**Fig 4**). In conclusion, by integrating the results of multiple prediction tools, EnsembleTFpredictor provides a set of biologically relevant candidate TFs ranked by confidence levels for easy prioritization for downstream validation and mechanistic studies.

### 3.4 Performance comparison on ChIP-seq data

**Fig 5:**
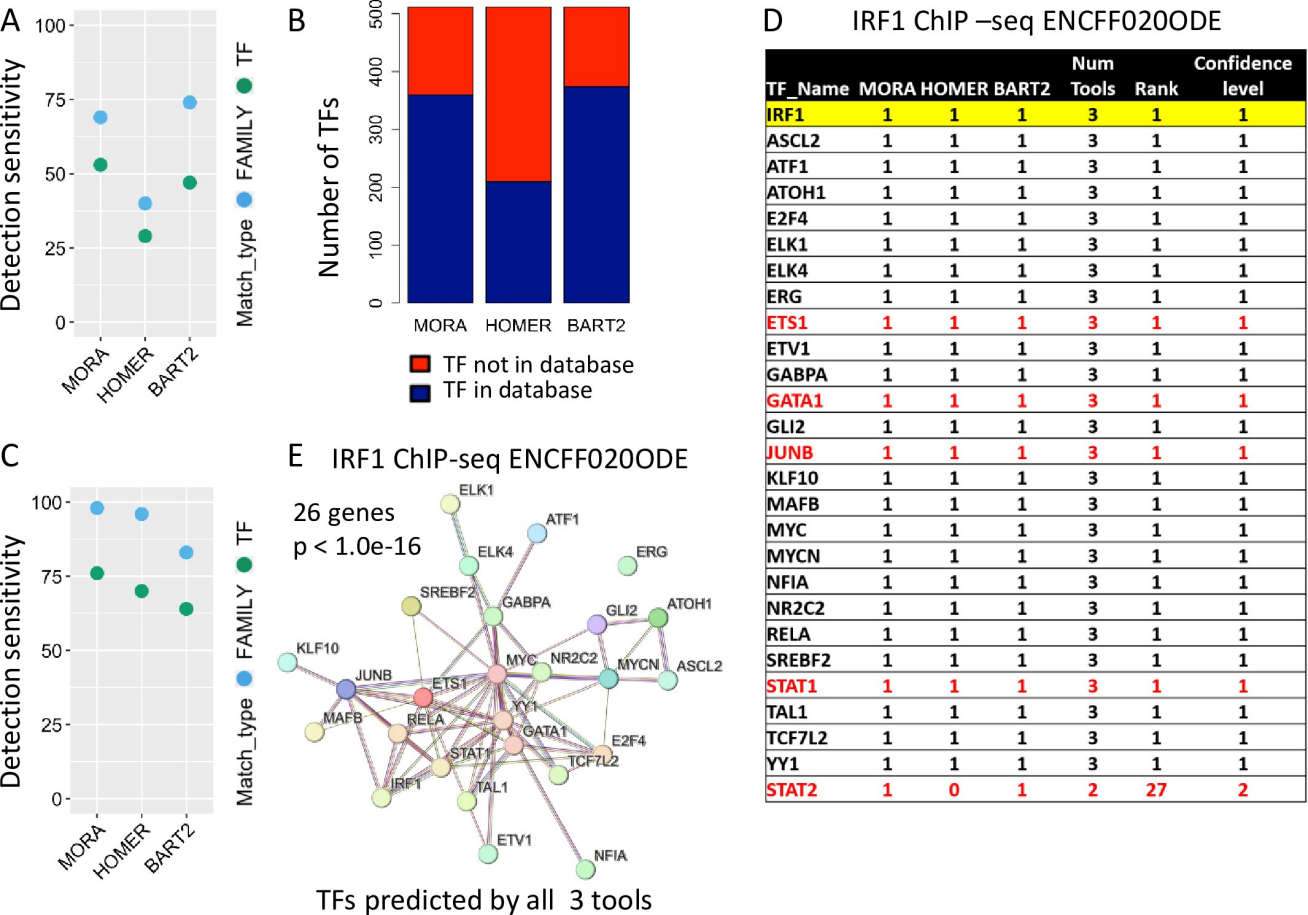
Performance evaluation of MORA and other published computational tools on ChIP-seq data. (A) Sensitivity plot for the ChIP-seq data target TFs showing detection sensitivity of each method at both the TF and family level. (B) Bar plot displaying the number of target TFs which are present/absent in the database of each method for the ChIP-seq data. (C) Sensitivity plot for the ChIP-seq target TFs including only those TFs which are in the corresponding method database. (D) Ranked list of identified TRs/TFs by prediction confidence level using EnsembleTFPredictor on ChIP-seq data. The TF highlighted yellow is the target TF. Red font indicates TFs that are known to interact with the target TFs. (E) Protein-protein interaction analysis results using STRING Database. TFs predicted by all 3 tools were used as the query gene sets for the STRING Database search.

Genomic regions derived from genomic or epigenomic profiling experiments such as ChIP-seq are commonly investigated in transcription regulation studies. The TF ChIP-seq peak data generated from the K562 cells were downloaded from the ENCODE portal [31] (https://www.encodeproject.org/), which included 512 peak files for 337 unique TFs at the time of download. The known target TFs for each dataset allow us to assess prediction accuracy at both the TF and family levels. MORA can be applied to any sequences without requiring additional information. MORA correctly predicted 52% (267/512) of the targets at the TF level, and 69% (352/512) at the family level (**Fig 5A and S21 Table in S1 Data**), with four samples failing to finish. HOMER, BART2, and LISA2 can also be applied to ChIP-seq data. LISA2 requires a list of genes in addition to a list of genomic regions, making it unsuitable for this analysis. We therefore compared the performance of HOMER and BART2 (**Fig 5A**) with that of MORA. HOMER had a lower sensitivity of 29% (147/512) and 40% (202/512) at the TF and family levels respectively (**S22 Table in S1 Data**), with one sample failing to finish. BART2 had a sensitivity similar to MORA with 49% (250/512) successfully predicted at the TF level, and 86% (438/512) at the family level (**S23 Table in S1 Data**). Out of the 512 TFs, 357 (69.7%) and 205 (40.0%) were correctly predicted by at least one or two methods, respectively (**S24 Table in S1 Data**).

The predictive power on ChIP-seq data is noticeably lower than that on the gene list data for all the methods tested. One possible reason for this is that the target TF information is not available in the current database used for prediction, making it impossible to accurately predict. We found that only 68% (350/512) of the target TFs have at least one PWM in the CIS-BP database (**Fig 5B**). Among this group of target TFs, MORA correctly predicted 76% (267/350) of the targets at the TF level and 98% (344/350) at the family level, similar to the results obtained using gene lists as inputs. HOMER, on the other hand, has a relatively small PWM database (450 mammalian PWMs representing 358 unique TFs) and includes only 41% (210/512) of the target TFs (**Fig 5B**). For this group of target TFs, HOMER achieved a prediction sensitivity of 70% (147/210) at the TF level and 96% (202/210) at the family level, which is comparable to the performance of MORA (**Fig 5C**). The BART2 database comprises over 7,000 human ChIP-seq datasets for 919 TFs/TRs with 374 target TFs present in this database. For this group of target TFs, BART2 had a prediction sensitivity of 67% (250/374) at the TF level, and 88% (328/374) at the family level. The sensitivity of all tools is much higher when the target TF information is available in the database. The larger PWM database used in MORA, along with its higher detection sensitivity compared to HOMER, enabled more accurate prediction of target TFs.

### 3.5 Ensemble approach on ChIP-seq data to define interactive regulatory networks

We generated multiple-tool ranked TF tables for each ChIP-seq data using EnsembleTFpredictor. Fifty-five out of the 512 target TFs were correctly predicted by all three methods, and 132 were predicted by at least two methods. We next evaluated the relationships between the target TFs and the other top-ranked predicted TFs. ENCFF020ODE is the interferon regulatory factor 1 (IRF1) ChIP-seq data from human K562 cells, a human immortalized myelogenous leukemia cell line, treated with Interferon gamma (IFN-γ). IFN-γ primarily signals through the Jak-Stat signaling pathway, dominated by the activity of the STAT1 homodimer, which functions together with interferon regulatory factors (IRFs) and NFκB to control transcription of target genes [53]. On the other hand, STAT2 can dimerize with STAT1 and substantially attenuate IFN-γ responses [54]. Twenty six TFs were predicted by all three tools, including the target IRF1 and STAT1, ranking number 1 in the list (**Fig 5D and S25 Table in S1 Data**). ETS1 and IRF1 together regulate hepatic stellate cell activation [55], whereas GATA1 and IRF1 together regulate megakaryocyte hyperproliferation [56]. Ectopic IRF1 expression can rescue Junb-deficient mice, suggesting that IRF1 functions downstream of JUNB in the liver [57]. STAT2 was predicted by MORA and BART2 but not HOMER. Multiple TFs known to function in response to IFN-γ were correctly predicted only by 1 or 2 tools, such as NFκB, SPI1, MAF, and JUN (**S25 Table in S1 Data**). The tissue-specific transcription factor Pu.1 (SPI1) allows for the IFN-γ-induced expression of CXCL9 in myeloid cells [58]. IFN-γ also represses “M2” gene expression in human macrophages by inducing coordinate suppression of binding by MAF to a subset of enhancers [59]. Additionally, a novel c-Jun-dependent signal transduction pathway was found necessary for the transcriptional activation of IFN-γ response genes [60]. KEGG pathway enrichment analysis using TFs predicted by all three tools (26 genes) identified the “Transcriptional misregulation in cancer” pathway as the enriched pathway (**Fig 3F**), which is consistent with the fact that the data sets were derived from a cancer cell line. Protein-protein interaction analysis of TFs predicted by all three tools using the STRING Database demonstrated highly significant protein-protein interactions among the TFs (**Fig 5E**). These results demonstrate the superior performance of MORA in identifying biologically relevant TFs and the power of EnsembleTFpredictor in defining cooperating transcription regulatory networks.

### 3.6 Application of MORA and EnsembleTFpredictor in guiding experimental design

MORA and EnsembleTFpredictor have been proven effective in identifying novel functional TFs. In a study performed by Chen *et al*., MORA and oPossum-3 (now discontinued) were used to identify TFs that regulate genes differentially expressed in the granule neuron precursors of conditional SnoN knockout animals compared with littermate control animals [23]. EnsembleTFpredictor identified five TFs predicted by both algorithms, and two of them, N-myc and Pax6, were shown to form physical complexes with SnoN to regulate granule neuron precursor development. In a second study, MORA, HOMER, and oPossum-3 were used to identify TFs that regulate genes differentially expressed during dorsal root ganglion (DRG) neuron axon regeneration [24]. EnsembleTFpredictor identified 43 TFs predicted by at least two methods, including known axon-regeneration TFs, as well as TFs not yet characterized in axon regeneration. Experimental testing of eight novel TFs demonstrated that a combination of *Ctcf* with *Yy1* or *E2f2* had a significant effect on regenerative axon growth, but not when *Ctcf*, *E2f2*, or *Yy1* were expressed alone. These publications highlight the value of MORA and EnsembleTFpredictor in guiding experimental design to identify novel TFs that cooperatively regulate transcription in different biological systems.

## 4 Discussion

EnsembleTFpredictor offers a new perspective of integrated functional TF prediction by leveraging several regulatory factor prediction software that have been developed. We provide the source code of EnsembleTFpredictor, making it easy to incorporate the prediction results of other tools. The current regulatory factor prediction tools have limitations because they rely on external databases containing existing knowledge from previous studies (*e*.*g*., PWMs or ChIP-seq data). This reliance on external databases compromises the sensitivity of these tools, particularly for TFs lacking PWMs or (epi)genomic data in the databases. In fact, only 59.6% (305/512) of the ChIP-seq target TFs were correctly predicted by at least one method (**S24 Table in S1 Data**). A more comprehensive database is needed to improve prediction sensitivity. By integrating the prediction results from multiple tools, EnsembleTFpredictor not only provides a confidence ranking of the predicted TFs but also generates a more comprehensive list of candidate regulatory factors compared to any individual tool. Candidate TFs predicted by a single tool can still be biologically significant, as demonstrated by the ChIP-seq target TFs and the known IFN-γ-response TFs predicted only by MORA, as described earlier. However, when multiple independent tools predict a given TF, it increases the likelihood that the TF is biologically relevant and warrants further validation and mechanistic investigation.

MORA is distinct from all other PWM-based tools in multiple aspects (S5 Table in S1 Data). First, the motif enrichment measurements and algorithms for enrichment calculation are distinct. MORA used the over-representation index (ORI) to measure motif enrichment, which takes into account both the number of motifs found in the sequences (motif density) and the proportion of sequences in which the motif is found. Homotypic clusters of TF binding sites (many adjacent TF binding sites for the same TF species) are prevalent in eukaryotic genomes, and they play an important role in gene regulation in the human and other vertebrate genomes [33, 34]. A one-tailed Student’s t-test was used to compare the ORI of the query sequences with the random sampled background sequences to determine enrichment. HOMER uses the ZOOPS model, which assumes that there is zero or one motif occurrence per sequence. It will not be able to capture the information in homotypic clusters of TF binding sites. It then uses the hypergeometric enrichment calculations (or binomial) to determine motif enrichment. AME [20] uses two scores for each sequence in computing motif enrichment: the ’PWM score’ is computed by scoring the sequence with the motif, and the ’FASTA score’ is either provided in the sequence header line or determined by the rank of the sequence within the sequence file. AME sorts the sequences in increasing order of FASTA score, and then ’partitions’ the sequences, labeling the first N sequences ’positive’ and the rest ’negative’. AME computes the significance of motif enrichment using these labels and the PWM scores. Then, it repeats the process using values of N from 1 to the total number of sequences and reports the partition with the highest significance. Pscan [19] computes for each input sequence a raw matching value, representing the likelihood for the TF to bind the promoter. It keeps as matching value the one corresponding to the highest-scoring oligo in each sequence and computes the mean of the matching value on the input sequence set. Then for each profile, the average matching score obtained from the input sequence sets are compared to the mean and standard deviation of the score on the whole genome promoter set. The over-representation or underrepresentation for each profile is finally assessed using a z-test, which associates with each profile the probability of obtaining the same score on a random sequence set. Second, the motif site representation and searching algorithms are different. MORA used the PATSER program [27], a widely used software distribution in the field, to determine TF binding sites based on a PWM. HOMER does not identify TF binding sites. Instead, it uses oligos of the desired motif length as a substitute to calculate motif enrichment [16]. AME does not determine a TF binding site. Instead, it “treat[s] each subsequence in the sequence as a possible match to the motif.” Pscan does not determine a TF binding motif either, but the algorithm “computes for each input sequence a raw matching value, representing the likelihood for the TF to bind the promoter, and keeps as the matching value the one corresponding to the highest-scoring oligo in each sequence”. Third, the background sequences used in the comparison differ. MORA uses random sampling of genomic sequences outside the region of interest or gene promoters of non-query genes, matching the number and length of query sequences as the background sequences. This process is repeated 100 times by default to consider variations in sequence characteristics during enrichment calculations. HOMER follows a similar approach but uses all promoters (except those chosen for analysis) as the background. AME uses shuffled input sequences as the background sequences. Pscan uses the whole genome promoter set as the control set sequence. All methods accept custom background sequences as input. Lastly, the statistics tests employed by each method to calculate enrichment are different (S5 Table in S1 Data).

MORA is the only tool that achieved 100% sensitivity at the TF family level when DEG gene sets were used as the query sequences. Since TFs in the same family tend to have similar binding specificity, the failure to report the target TF for some datasets could simply be because the specific target TF does not have a representative PWM in the CIS-BP database. When ChIP-seq peaks were used as the query sequences, MORA correctly predicted 69% (352/512) of target TFs at the family level. However, only 68% (350/512) of the target TFs have PWMs present in the CIS-BP database (**Fig 5B**). If we focus on this group of TFs MORA had a sensitivity of 98% (344/350) at the family level. This suggests that the major reason we failed to report a target TF for a gene set is because the target TF does not have a representative PWM in the database. Since CIS-BP is one of the most comprehensive publicly available motif databases, offering CIS-BP as a database choice by HOMER and Pscan will likely improve the prediction performance. An alternative reason that a target TF was not reported could be that the query sequences are not the primary regulatory targets of the TF but rather an indirect effect via other TFs. This seems to contribute only to a small fraction of the cases, if at all.

## 5 Conclusion

All the computational tools tested in this study can predict biologically relevant TFs. In terms of sensitivity, MORA outperforms all the PWM-based tools and is comparable to the (epi)genomic-data-based tools. Most importantly, by harnessing the predictive power of all the tools, EnsembleTFpredictor provides a high-confidence candidate TF list and has proven to be useful in identifying functional and cooperative regulatory TFs in multiple biological systems. The minimum number of tools required as inputs for EnsembleTFpredictor is two. Because TFs predicted by algorithms with completely different underlying mechanisms are more likely to be functional, we recommend using at least one tool from each category; MORA from the PWM-based tools and either BART2 or Lisa2 from the (epi)genomic-data-based tools. BART2 or Lisa2 typically predicts hundreds of candidate TFs, whereas HOMER predicts a much shorter list of candidate TFs. Including HOMER could help narrow down the candidate TFs for downstream validation. Furthermore, MORA and EnsembleTFpredictor can be applied to any type of genomic sequences, such as those obtained from the assay for transposase-accessible chromatin with sequencing (ATAC-Seq), DNase I hypersensitivity assay, or histone modification ChIP-seq assay, making them a truly invaluable resource for the wider scientific community.

## Supporting information

S1 Data This is the supplemental table including S1–S25 Tables. (XLSX)
